# Illicit tobacco trade is ‘booming’: UK newspaper coverage of data funded by transnational tobacco companies

**DOI:** 10.1136/tobaccocontrol-2018-054902

**Published:** 2020-04-16

**Authors:** Karen Evans-Reeves, Jenny Hatchard, Andy Rowell, Anna B Gilmore

**Affiliations:** Tobacco Control Research Group, Department for Health, University of Bath, Bath, UK

**Keywords:** Illegal tobacco products, Media, Tobacco industry

## Abstract

**Background:**

Transnational tobacco companies (TTCs) have heavily publicised their argument that standardised tobacco packaging will increase the illicit tobacco trade. Leaked Philip Morris International (PMI) documents suggest that the company may have intended to use third parties to promulgate this argument in the UK.

**Methods:**

We examined articles in UK newspapers (1 April 2013 to 31 March 2015) from LexisNexis for presence and nature of tobacco industry data. We also examined documents released by Freedom of Information requests made to Scottish Councils for evidence of how PMI operationalised its third-party strategy.

**Findings:**

Two-thirds of newspaper articles (63%, 99/157) mentioned a PMI consultant; 36% of which did not disclose this industry funding. Most articles mentioned counterfeit tobacco, illicit whites or both (72%, 113/157), while few (4%, 7/157) specifically mentioned tobacco industry illicit tobacco and none explained that the latter can include tobacco-company involvement. Freedom of Information documents revealed that the PMI consultant sought to build relationships with Trading Standards officers, conducted undercover test purchases (UTPs) in illicit tobacco ‘hotspots’ and may have promoted unrepresentative findings in the media. While the data set featured PMI data predominantly, other TTCs also engaged in third-party techniques to promulgate messages on illicit tobacco.

**Interpretation:**

PMI engaged a third party, seemingly with the aim of securing media coverage on illicit tobacco positing that standardised packaging would worsen the problem. The predominant focus of articles which featured industry-funded data and information was on counterfeit tobacco despite official data showing tobacco-industry illicit tobacco as the most prevalent. Other jurisdictions considering the policy should anticipate that third parties will promote the illicit-trade argument.

## Introduction

Official UK figures show a downward trend in the illicit tobacco market from 10 billion cigarettes in 2005/2006 to 5.5 billion in 2016/2017.^1^ Similarly, there has been a decrease in illicit hand-rolled tobacco from 800 000 kilos to 500 000 kilos over the same time period. Despite this downward trend in illicit tobacco, transnational tobacco companies (TTCs) continue to oppose tobacco-control policies, arguing that these policies will increase the illicit trade in tobacco products.^2 3^ They make such arguments despite extensive historical^4–7^ and some contemporary evidence^8^ of TTC involvement or at least awareness of the smuggling of their own products (tobacco-industry illicit).

TTCs fuel this debate by publicising the findings of research that they have commissioned.^9 10^ For example, in 2011, when illicit cigarette volumes were at their lowest in the UK (3 billion sticks), TTC-funded data on illicit tobacco started to appear in the UK press, with stories reporting increases in illicit trade that were not supported by independent data.^11^ This proliferation of stories coincided with the UK Government’s announcement that it intended to hold a public consultation on the standardised packaging of tobacco products (box 1). This activity has been viewed as an attempt to interfere with the progression of the policy in the UK.^11^ Furthermore, a recent systematic review revealed that industry-funded data on illicit trade are methodologically opaque and tend to overestimate its scale in comparison to independent data.^12^

Box 1 **Standardised packaging**

- Standardised packaging legislation, which was introduced in Australia in December 2012 and in France and the UK in May 2016, requires all design branding to be removed from cigarette and roll-your-own tobacco packets, restricting the colour of each pack to Pantone colour 448 C (often referred to as ‘drab’ green) and homogenising the type face and font size of the brand name and variant.^41^ The Canadian Cancer Council produces a regular update, listing all countries that have implemented or are in the process of implementing standardised packaging.^42^

In addition to overestimating the scale of the problem, TTCs have overestimated levels of counterfeit and illicit whites (table 1 for definitions of the different types of illicit tobacco).^10 12^ Yet, previous research (including industry-funded research),^13–19^ has consistently shown that the highest proportion of the illicit-tobacco market is made up by tobacco-industry illicit tobacco, which suggests that TTCs are culpable to some extent.^10 20^ In 2014, during the timeframe of this study, Operation Henry data, produced by the Trading Standards Institute (a not-for-profit organisation that supports Trading Standards and involves the collection of seizure data), revealed that 72% of illicit cigarettes seized were tobacco industry illicit, 24% were illicit whites and just 5% were counterfeit cigarettes.^20^

**Table 1:** Definitions of different terms used to describe the illegal tobacco trade and duty-free sales

Terminology	Usage	Definition
Illicit trade	General term encompassing all illegal forms of tobacco	Illicit trade is defined in Article 1 of the WHO’s framework convention on tobacco control as ‘any practice or conduct prohibited by law and which relates to production, shipment, receipt, possession, distribution, sale or purchase including any practice or conduct intended to facilitate such activity’.^43^ Includes smuggled genuine tobacco (tobacco industry illicit), counterfeit tobacco and ‘Cheap/illicit Whites’.
Non-domestic product (known in the UK as non-UK duty paid)	General term encompassing illegal and legal tobacco	Tobacco on which local (eg, UK) duties have not been paid, either illegally or legally in compliance with regulations concerning transport of tobacco products between jurisdictions. The latter includes cross-border sales (which in turn include duty free – see below).
Smuggled	General term for non-domestic illegal tobacco	Tobacco which has been brought into the country illegally without paying local duties. Can include counterfeit, illicit whites and tobacco-industry illicit tobacco. Does not include legal cross-border or duty-free sales.
Contraband	General term but is sometimes used as a specific term.	General: comprises tobacco sold in violation of applicable duty (includes counterfeit and tobacco-industry illicit tobacco). Specific: genuine tobacco produced by licenced manufacturers but local duties have not been paid (tobacco-industry illicit tobacco).
Tobacco-industry illicit	Specific term	Genuine tobacco produced by licenced manufacturers but local duties have not been paid.
Cheap whites/illicit whites	Specific term	Illicit whites are cigarettes manufactured for the sole purpose of being smuggled into and sold illegally in another market. They usually do not pay tax in the country where they are made.^44^
Cross-border sales/shopping	Specific term	Legal importation of goods for personal use. Includes duty-free cigarettes/tobacco and those, in the case of the UK, with duties paid outside the UK, eg, in other EU countries.
Counterfeit	Specific term	Products bearing a trademark of a tobacco manufacturer which are manufactured by a third party without the consent of the manufacturer.^45^

In 2013, a leaked Philip Morris International (PMI) corporate affairs presentation revealed that the company intended to use illicit trade as a counterargument to standardised packaging to ensure that the policy ‘was not adopted in the UK’.^21^ PMI intended to raise awareness of the illicit-trade argument among ‘decision makers and the general public’ by using ‘third-party media engagement’ and ‘high-profile opinion pieces’, and identified two third-party media messengers that it would use to do so.^22^ First, the Anti-Counterfeiting Group, a trade association campaigning against imitation products with TTC fee-paying members. Second, the initials of a PMI consultant, a retired police officer engaged by PMI since November 2011 to, in his own words, ‘conduct extensive research into the illicit trade in tobacco products and to act as a spokesperson on the subject’.^23^

Given that standardised packaging for tobacco products is now being considered globally and that tobacco-industry data on illicit tobacco have been shown to exaggerate the scale of the problem,^12^ the objectives of this study were three-fold. First, to explore whether and how UK newspapers presented TTC data on illicit tobacco. Second, to understand the extent to which PMI operationalised its proposed use of third-party media engagement and the illicit-trade argument. Third, to assess whether articles were accurately reporting financial losses to the Exchequer resulting from the illicit-tobacco trade.

## Methods

### Data collection

English-language UK newspaper articles between 1 April 2013 and 31 March 2015 obtained from the LexisNexis database were examined for presence, nature and timing of TTC data measuring illicit tobacco in the UK or overseas. Search terms in Lexis included combinations of ‘illegal’, ‘illicit’, ‘smuggling’, ‘tobacco’, ‘cigarette’ and all four TTCs operating in the UK, Imperial Tobacco (IMT), Japan Tobacco International (JTI), PMI and British American Tobacco (BAT). In addition, from the intelligence gained from PMI leaked documents we searched for both the PMI consultant and the Anti-Counterfeiting Group alongside ‘illicit’ or ‘illegal’, both with and without the names of the four tobacco companies.

In total, searches returned 428 newspaper articles.

### Inclusion criteria

Articles were included if they referred to specific industry data, methods or reports and excluded if they were duplicates or made general statements such as ‘illicit tobacco has increased’ but provided no data. Identical articles published on different days in the same newspaper and identical articles published in different newspaper titles were included. KER and AR double coded all articles for relevance using the inclusion and exclusion criteria.

### Data coding

Our coding framework was part deductive, based on prior work,^11^ and part inductive, based on examination of 10% of the articles, to further develop the framework. We coded the data contained in each article by tobacco company responsible for the data (eg, explicitly named as the data funder or implicated through a known third party), any mention of a third party involved in the creation of the data, illicit-tobacco terminology used (we recorded all terms used; table 1), methodology used to collect the data, geography of the data (ie, which country the data referred to), whether it was published in a subnational or national newspaper and whether or not official government departments were mentioned in the articles. (table 2 for definitions of all other variable and category descriptions) KER and JH primary coded 50% of the data each and second coded 10% of each other’s data. All discrepancies were resolved.

**Table 2:** Number of newspaper articles stratified by characteristics of interest (n=157)

Category	Explanation of terms	Tobacco company/companies responsible for the data						Total
		PMI	IMT	BAT	JTI	Other*	Consultant & not PMI	
**TTC third party involved in data creation†**								
PMI consultant	Consultant hired by PMI to conduct UTPs	53	0	0	0	10	36	99
Anti-counterfeiting group	Campaign group co-founded by the tobacco industry with tobacco-industry members	0	0	0	0	0	0	0
MS Intelligence	Research consultancy hired by PMI to conduct EPSs	5	0	1	0	2	7	15
KPMG	Consultancy hired by PMI (& more recently other TTCs) to compile data from EPSs	6	0	0	0	8	2	16
Populus	Research consultancy hired by PMI to conduct surveys	3	0	0	0	0	0	3
Newspaper collaboration	TTC working together with a newspaper on seizures	0	1	0	8	0	0	9
Royal United Services Institute for Defence and Security Studies	Research consultancy hired by PMI	1	0	0	0	0	0	1
Tobacco Manufacturers Association/Tobacco Retailers Alliance	Organisations wholly owned by TTCs to campaign on their behalf	0	1	1	0	6	0	8
**Type of Illicit mentioned†**								
Smuggled	See table 1 for full definitions	36	5	0	18	13	28	100
NUKDP		9	3	0	8	4	10	34
Contraband		19	2	0	4	2	7	34
Counterfeit/fake		48	9	0	17	5	31	110
Tobacco-industry illicit (the term ‘genuine’ product used in the article)		5	0	0	1	0	1	7
Cheap/illicit whites		22	1	0	7	1	8	39
Cross border sales		3	1	0	4	4	7	19
**Data methodology†**								
Undercover test purchases	Attempts to purchase illicit tobacco without disclosing the true purpose of the purchase	47	0	0	14	3	26	90
Empty pack survey	Collection of discarded packs which are assessed as domestic or non-domestic	16	1	1	1	12	8	39
Raid/seizure	A search of premises usually in collaboration with trading standards	0	8	0	2	0	1	11
Other methodology‡	Any other methodology including surveys or interviews conducted with tobacco retailers for their perceptions on the scale of illicit trade	9	5	0	9	5	9	37
**Data nationality**								
UK	TTC data refers to the UK	57	13	1	22	11	39	143
Australia	TTC data refers to Australia	1	0	0	0	8	2	11
Both	Article includes TTC data from both UK & Australia	3	0	0	0	0	0	3
**Newspaper**								
Subnational	Article appeared in a subnational publication	47	12	0	16	9	37	121
National	Article appeared in a national UK publication	14	1	1	6	10	4	36
**Government agency mentioned§**								
HMRC only	HMRC	13	1	0	5	5	11	35
Trading standards only	Trading standards agency	17	8	0	1	2	11	39
Both	Both HMRC & trading standards mentioned in the same article	12	2	0	7	1	0	22

*Includes where ‘tobacco companies’ in general are mentioned as the data source; two or more tobacco companies are named; or the tobacco industry owned Tobacco Manufacturers Association is attributed as the data source.

†Will not add up to 157 as more than one type of data collection method was mentioned and more than one type of illicit tobacco was mentioned in many of the articles or sometimes TTCs presented their own data; that is, there was not always a third-party involved in the creation of the data.

‡Other methodology includes TTC funded surveys of retailers, police officers and other interested parties’ opinions on the scale of illicit tobacco and data provided by tobacco companies and industry data of unknown methodology.

§Will not add up to 157 as only 96 articles mentioned a Government agency.

BAT, British American Tobacco; EPSs, empty-pack surveys; HMRC, Her Majesty’s Revenue and Customs; IMT, Imperial Tobacco; JTI, Japan Tobacco International; NUKDP, non-UK duty paid; PMI, Philip Morris International; TTCs, transnational tobacco companies; UTPs, undercover test purchases.

### Data analysis

#### Newspapers

We conducted a quantitative content analysis of the newspaper articles to categorise and assess the characteristics of industry data on illicit tobacco (eg, data-collection method, third-party involvement in its creation and type of illicit tobacco presented), by TTC data source.

In some instances, articles noted the amount of revenue lost to Her Majesty’s Revenue and Customs (HMRC) due to illicit trade. We compared these amounts with the official HMRC data.^24–26^

Additionally, we compared the proportion of articles that included mentions of particular types of illicit tobacco (eg, counterfeit, illicit whites, tobacco-industry illicit, etc) with independent data from the 2014 Trading Standards Institute Operation Henry report^20^ which detailed the most prevalent forms of illicit tobacco seized in the UK at that time.

#### Freedom of information documents

To explore the role of the PMI consultant identified in the PMI leaked documents, we analysed documents released from Freedom of Information (FOI) requests made by Action on Smoking and Health (ASH) Scotland to each of the 32 Scottish Councils’ Trading Standards teams. The FOIs requested information relating to any contact between the Council and the consultant, or anyone acting on his behalf; from the beginning of 2011 to March-May 2014, including all communication, details and minutes of meetings and any other documents which refer to them and/or to their activity on illicit tobacco. We conducted a qualitative thematic analysis of the FOI documents using NVivo 10 software to assess the purpose of the communication between the two parties. After we identified the main purposes of the communication, we selected illustrative quotes to support our interpretations and collated them in a table. We compared any corresponding media coverage from our newspaper data set to check the congruity between what the consultant reported to Trading Standards and the content of newspaper articles.

## Results

### Press coverage characteristics

Between April 2013 and the end of March 2015, 157 media articles contained tobacco industry data on the illicit-tobacco trade, four were published letters, one was an opinion piece presenting the two opposing sides of the standardised packaging debate and the remainder were newspaper articles.

PMI funded the data in 65% (107/157) of the articles, JTI 14%, IMT 8% and BAT<1%. All four TTCs, a combination of two or more TTCs, known tobacco-industry entities (eg, the Tobacco Manufacturers Association, Tobacco Retailers Alliance), or just ‘tobacco companies’ in general funded the data in the remaining 12% of articles. Although we were able to attribute 65% of articles to PMI, 34% (36/107) of these articles did not openly disclose PMI as the data funder (table 2). Each of these 36 articles mentioned a known PMI consultant but did not mention PMI or any other tobacco company. In total 99 articles included the consultant and an additional eight articles mentioned PMI data without the consultant.

In addition to the PMI consultant, articles reported that nine other third-party groups were involved in the creation and analyses of the data (table 2). Four of these have been contracted by PMI. *MS Intelligence*, a research consultancy hired to conduct empty-pack surveys^27^; *KPMG* who compiled data from empty-pack surveys (more recently other tobacco companies have also funded this work),^27^ *Royal United Services for Defence and Security (RUSI*) was contracted to conduct research on illicit tobacco and organised crime,^28^ and *Populus* who conducted opinion surveys.^9^ JTI conducted investigations in collaboration with *Crimestoppers* as well as with two newspapers, *The Argus* and *The Manchester Evening News* and IMT did the same with *The Burton Mail*. IMT attended raids alongside Trading Standards officers and sniffer-dog company Wagtail. It is not clear whether IMT paid for the sniffer dogs in these instances but we do know that they have offered to pay for sniffer dogs previously.^29^

#### Types of illicit tobacco mentioned in the articles and clarity of definitions

The majority of articles (77%) used other terms beyond illicit, illegal or smuggled tobacco (that were in the search terms for articles). Additional terms included counterfeit/fake, contraband, non-UK duty paid, cheap/illicit whites, cross-border sales and other words to describe tobacco-industry illicit such as ‘genuine’ product. Some terms were used more frequently than others. Over two-thirds (70%) mentioned counterfeit or ‘fake’ tobacco, 64% mentioned smuggled tobacco, 25% mentioned illicit whites, 22% mentioned contraband and just 4% specifically referred to genuine tobacco-industry product sold illegally (table 2).

Smuggled tobacco can include both counterfeit, illicit whites and tobacco-industry illicit (or contraband). However, in those 64% of articles that mentioned smuggled, over three-quarters of these also mentioned counterfeit or fakes, whereas just under one-third mentioned illicit whites while less than one-quarter mentioned contraband (which is sometimes used to describe genuine tobacco-industry illicit) and just seven (4%) specifically mentioned the word ‘genuine’ tobacco-industry product. Therefore, counterfeit tobacco was discussed most in news articles about smuggled tobacco. Of those mentioning contraband, half used it as a general term for illicit tobacco while the other half referred to contraband in addition to counterfeit tobacco, which therefore suggests that there is a distinction between the two terms. However, just one article which made this distinction explained that contraband can include tobacco-industry genuine smuggled product. None of the articles mentioned the tobacco industry’s potential complicity in the smuggling of its own products.

#### Underlying data methodology

Over half of the articles (57%) included data gathered using undercover test purchases (UTPs), 25% used empty-pack surveys (EPSs). The remainder cited data from raids, seizures or other methodologies. In 13% of articles more than one data-collection methodology was discussed, for example, UTP and EPS.

#### Government departments

Over half (61%) of the articles mentioned either HMRC or Trading Standards in some capacity, mentioning either data or quotes from the agencies, or simply reporting that the industry-funded data had been handed over to these agencies.

##### ​Data

Two-thirds of the articles that cited HMRC data were either about illicit tobacco or the amount of money lost to the Treasury as a result of illicit tobacco sales. Half of these (n*=*29) referred to HMRC’s top-end estimate (or higher) of the amount of revenue lost to the Exchequer (between £2.8 billion (bn) and £3.6bn),^26^ which overestimated the scale and impact of the illicit-tobacco trade. This was significantly more than the HMRC-recommended mid-estimate of £2bn. This midpoint figure has since been revised to £2.2bn.^1^

##### ​Collaboration

In 15% of articles, interactions between the tobacco industry and one, or both, of the government agencies, were reported, ranging from raids on premises suspected of supplying illicit tobacco, to sharing intelligence on ‘illicit hotspots’.

##### ​Information has been shared

One-third (34%) stated that the industry data was given to either HMRC or Trading Standards for further investigation.

##### ​Quoted

Finally, HMRC (15%, 14/96) and Trading Standards (25%, 24/96) were quoted in these articles. Most quotes talked about illicit tobacco in general terms and did not refer to any working relationship or correspondence with TTCs nor did they make any mention of any tobacco-industry third party or their data.

### PMI consultant: Freedom of Information documents

Twelve out of thirty-two Scottish Councils confirmed that Trading Standards officers had met the PMI consultant to discuss illicit tobacco (table 3); eight released their correspondence, three refused access to documents and one council said no documentation was available. A further two councils refused to either confirm or deny any interactions and the remainder reported no record of any correspondence.

**Table 3:** Illustrative quotes from FOI documents that illustrate the purpose of PMI’s UTPs in Scottish Councils

	Quote	Council	Date
**1. Building relationships & influencing perceptions**			
Meetings & presentations	‘He is meeting XXXXXX in Aberdeen on Thursday at 11 am to discuss illicit cigarettes. Wants to know if Shire (Aberdeenshire) ‘want to do similar’. His number is XXXXX. Can you give him a call to attend the meeting or set up a separate meeting?’	Aberdeenshire	16 Sept 2013
Offering training on illicit	*‘The Tobacco Co that I couldn’t remember the name of last week when I was speaking to you is Philip Morris. Apparently XXXX can offer assistance and training! Think one of the Ayrshires was using him as he can provide tobacco readers. I will find out next meeting what he does and exactly how good he was!’* (email between two Trading Standards officers)	Renfrewshire	18 Sept 2013
Information sharing (not necessarily actionable)	*‘I did explain to XXXX that unfortunately our test purchase work in the UK in general is only for intelligence. I am sorry about that but it is a company policy.’* (PMI consultant email to trading standards officer)	Stirling and	22 Nov 2013
	*‘In order for us to follow-up the information we received last week via Falkirk Council Trading Standards Service relating to the Alloa premises we need to seek a warrant for the private residence. For us to do this we require rather more information than ‘TP met XXXX, XXXX took him to private address and sold’ or ‘Met male in bar said he could supply, product arrived by car later.’’* (email from Trading Standards officer to PMI consultant)	Clackmannanshire	25 Nov 2013
**2. Information seeking**	*‘Coming up to Fife next week – spend 2–3 days here. XXXX noted down a couple of areas/towns that TS feels could be potential problems, however can also email XXXX re any shops etc we feels* (sic) *may be of interest. XXXX will provide feedback to TS on anything of relevance found*.’ (trading standards minutes of meeting with PMI consultant)	Fife	13 Nov 2013
	*‘Any help you can give us as to likely areas or locations would be appreciated.’* (Email from PMI consultant to Trading Standards officer)	Renfrewshire	23 Oct 2013
	*‘I appreciate that it is a very busy time for you but any pointers for the guys would be useful.’* (email from PMI consultant to Trading Standards officer)	Renfrewshire	28 Oct 2013
**3. Creating publicity**	*‘…our local press have contacted the Council regarding what action we are taking at the above market (Ayr Sunday market). They say they have been contacted by a company to say illicit sales are taking place at the market…. Is there someone in your organisation that could update us on what line you have taking* (sic) *in your release to the papers?’* (email from trading standards officer to PMI consultant)	South Ayrshire	Nov 2013 (exact date unclear)
	*‘I have been forwarded your email addressed to XXXX, who is our consultant on the illicit trade. Please find attached our press releases for this area.’* (email from PMI staff, illicit trade strategies & prevention to Trading Standards officer)	South Ayrshire	27 Nov 2013
**4. Overestimating the scale of the problem**			
‘Illicit tobacco trade is booming’ (incongruous statements to Trading Standards and the press)’	*‘Ayr was by far the lowest amount of buys we have had so far in over 20 towns/cities in the UK.’* (email from PMI consultant to trading standards officer)	South Ayrshire	16 Oct 2013
	*‘Ayr was one of the areas in which we found it easy to access… We found that the illicit tobacco trade in Ayr is booming.’* (PMI consultant quote in “Terrorist Cigs Link; Loyalists in smokes racket”, *Scottish Star*)		2 Dec 2013

FOI, Freedom of Information; PMI, Philip Morris International; TS, Trading Standards Agency; UTPs, undercover test purchases.

The documents revealed that the consultant and a team of associates had conducted numerous UTPs in Scotland between October 2013 and November 2013. Our press data set revealed 27 regional and national (Scotland) media articles from October 2013, which corresponded with these UTPs. Eight presented both the PMI UTPs and a critique of them from either a Non-Government Organisation (eg, ASH Scotland) or a tobacco-control expert, while the majority presented just the PMI UTPs without any comment from a public health organisation or individual.

Thematic analysis of the documents suggests four main facets of the PMI consultant’s activities (illustrative quotes from the FOI documents are provided for each in table 3):

#### Building relationships and influencing perceptions

The consultant attempted to build relationships with Trading Standards officers using three main techniques. Namely, (i) setting up meetings with Trading Standards and giving presentations on illicit tobacco, (ii) offering training on illicit tobacco, (iii) sharing information on test purchases conducted in each Council.

**Figure 1:**
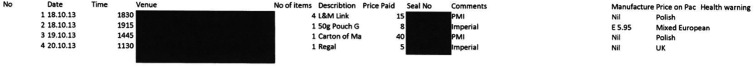
Spreadsheet of undercover test purchase intelligence conducted by the PMI consultant in October 2013 and provided to the Scottish borders trading standards team. PMI, Philip Morris International.

Relating to the latter, it is not clear whether the intelligence provided was useful or enabled any successful legal action to be taken. Although at least seven Councils received an Excel spreadsheet of intelligence from the UTPs, all but one of these were redacted from the FOI releases. The released spreadsheet reported seven purchases of illicit-tobacco items but it is not clear from the descriptions whether the tobacco was tobacco-industry illicit, counterfeit, illicit whites, etc (figure 1). Furthermore, the FOI release from Stirling and Clackmannanshire revealed that the Council was not able to take action based on the limited information provided by the consultant on his UTPs. When the Council requested further information, the consultant responded that no further information would be provided. (table 3) This apparent limited utility contrasts with the way the PMI consultant presented the UTPs in the press articles:

‘All the information we have has been fed back to Trading Standards, and I know they’ve done seizures and searches. We hope we assist law enforcement agencies to get this tobacco off the street – and get the people responsible.’^30^

#### Information seeking

As well as providing information to Trading Standards, the consultant sought information from Councils in order to target UTPs in illicit ‘hotspots’, rather than taking a random sample of stores and other locations to test the availability of illicit tobacco.

#### Creating publicity

In its leaked corporate affairs plan,^21^ PMI was explicit that, to oppose the implementation of standardised packaging in the UK, an integral part of the company’s strategy was to publicise the findings of UTPs in the press. To this end, the PMI consultant appeared to have direct support from PMI. Correspondence in the FOI documents suggests that the company drafted at least one press release for local media outlets about the UTPs in Scotland (see table 3 for a quote supporting this statement).

#### Overestimating the scale of the problem

Given that illicit-tobacco ‘hotspots’ were sought, the small number of purchases recorded and the brevity of the intelligence provided to Trading Standards (figure 1), the information was seemingly unrepresentative of each Council area. Nevertheless, this information was often extrapolated in the media to make generalised claims about the proportion of illicit tobacco in each town/city/council. (see online supplementary table 1) The press coverage of the Scottish UTPs in the 27 articles identified illicit tobacco at a town, city or council level was a significant and rising problem using terms such as *rife*, *booming*, ‘*flooded with fakes*’(online supplementary table 1). Articles also claimed that illicit tobacco had doubled or increased by one-third since the previous year. Furthermore, in at least one case there was a mismatch between what the press coverage claimed and the information provided to the Councils.

10.1136/tobaccocontrol-2018-054902.supp1 Supplementary data

For example, in Ayr, the PMI consultant was quoted in the *Scottish Star,* saying that ‘the illicit tobacco trade in Ayr is booming’, yet at the same time the consultant’s feedback to South Ayrshire Council suggested that the consultant and his team had found it difficult to locate illicit tobacco in Ayr with the ‘lowest amount of buys we have had so far in over 20 towns/cities in the UK.’(table 3)

## DISCUSSION

In the 2 years that preceded the March 2015 Parliamentary vote in favour of introducing standardised tobacco packaging in the UK, tobacco-industry data featured heavily in the UK regional press. Despite PMI’s relatively low market share of 9%, two-thirds of the newspaper articles in this study included the company’s data on illicit tobacco. Consistent with leaked documents detailing its anti-standardised packaging strategy, PMI engaged third-party media messengers including the consultant referred to in this paper, and research consultants *KPMG*, *MS Intelligence, RUSI* and *Populus,* to conduct research into, and promulgate their findings on, the illicit-tobacco trade. (figure 1) In over one-third of the PMI-linked articles that mentioned the consultant, only the consultant’s previous position as a former police officer was mentioned, readers were not informed of the consultant’s financial link to PMI. It is unclear who was culpable for this omission — the publication editors or the consultant. However, four were seemingly self-penned letters, signed by the consultant and published without any mention of PMI.^31–34^

The documents released under FOI legislation revealed the four key facets of the PMI consultant’s activities: relationship building, information seeking, creating publicity and overestimating the scale of the problem. First, the consultant sought to build relationships with Trading Standards officers in Scottish Councils through emails and meetings in order to acquire intelligence, in particular about the location of retailers most likely to sell illicit tobacco. While UTPs were then seemingly only attempted in these preidentified locations, the estimates used in subsequent publicity extrapolated the UTP findings to the entire town, city or council. This sampling methodology was not hidden from the public (eg, PMI press release circulated to the Scottish Economy Committee,^35^ Home Affairs Select Committee inquiry into tobacco smuggling^23^ and some of the media articles included in this study), but this methodology has been criticised for selective bias.^36^ Furthermore, the majority of articles present findings favourable to the industry, stating illicit tobacco is increasing and highlighting counterfeited tobacco where the industry is portrayed as the victim, over tobacco-industry illicit tobacco, which points towards potential industry complicity.

In comparison, only seven articles talked about genuine smuggled product. However, not one article mentioned that the presence of tobacco-industry illicit might implicate tobacco company involvement or at least lack of sufficient diligence in their management of their supply chains.

The majority of articles did not make it clear that smuggled, contraband and non-duty-paid tobacco can include tobacco-industry illicit as well as counterfeit. This focus on counterfeit tobacco and ambiguity about what exactly smuggled and contraband tobacco are does not reflect the official figures of the illicit-tobacco market in the UK at the time of the study. According to both industry and independent data, the majority of illicit tobacco was tobacco industry illicit with only a very small proportion attributable to counterfeit.^10 20^ Therefore, in this respect, the media coverage generated by tobacco companies in the study period can be considered misleading.

Furthermore, although in some cases, articles accurately cited HMRC’s mid estimate of £2.1bn as the potential cost of illicit tobacco to the Exchequer, 29 articles (18%) included figures far in excess of this, predominantly the top estimate of £2.9bn, with some stating £3bn and others as much as £3.6bn. JTI was reprimanded by the Advertising Standards Authority for citing this £3bn figure in anti-standardised packaging adverts that the Company had placed in UK broadsheet newspapers in 2012.^37^ No articles in this study presented the lower HMRC estimates.

Given that 61% of the media articles mentioned either Trading Standards or HMRC, readers may reasonably assume (whether it was the case or not) that TTCs and official government agencies were cooperating and that the purpose of the UTPs was to provide actionable intelligence to Trading Standards to help reduce illicit trade. However, at least one document in our data set suggests that the information provided to Trading Standards by the consultant was not actionable and the consultant said that the information was ‘only for intelligence’ and that this was ‘company policy’. Since this time, correspondence with several Trading Standards teams in Scotland revealed that the intelligence provided by the PMI consultant had not led to successful prosecutions. Not all Councils responded to requests for information. However, none responded with information on any legal action taken. HMRC said its policy was not to share any information held.

Although PMI was responsible for the data in over half of the articles during the study period, IMT, JTI and BAT were all attempting to raise public concern about illicit tobacco. JTI collaborated with Crimestoppers (sparking controversy over undue industry influence)^38^ and subnational press on UTPs. IMT involved themselves in Trading Standards raids, alongside sniffer dogs (it is not known if IMT providing the funding for these), and BAT was involved in EPS studies.

### Limitations

The extent to which TTCs were able to influence the content of newspaper articles in national and regional publications is not quantified in this study. Attempts by the authors to speak to the editors of many of the publications were unsuccessful. The relationship between the media and their information sources, particularly those with a potential conflict of interest, requires further investigation. However, it is clear that some publications, such as *The Argus*, *The Manchester News* and *The Burton Mail* took part in operations by JTI and IMT and subsequently published newspaper articles. Furthermore, there is evidence that in at least one Council in Scotland, PMI wrote a press release of the consultant’s UTPs. It is unclear whether this release was published in its entirety, in part or not at all.

We conducted our searches for relevant articles in LexisNexis. Unfortunately, this database does not include all national and subnational press publications and so it is highly likely that we have underestimated the number of press stories containing tobacco-company data on the illicit-tobacco trade.

During the study period, following a legal agreement with the European Union (EU), PMI was obliged to produce a yearly report on the illicit trade in EU member states.^27^ PMI contracted KPMG (who subcontracted MS Intelligence) to collect data and produce reports. However, 99/157 articles mention the consultant, in comparison to the 31/157 that mentioned KPMG or MS Intelligence. Therefore, PMI’s media coverage on this topic cannot be solely explained by its EU obligations.

Finally, this paper focused on mainstream media and did not include other forms of information dissemination such as social media.

## CONCLUSION

This study contributes to the growing body of research which suggests TTCs oppose tobacco control policies by, among other things, constructing and promoting a narrative of a dysfunctional future, where the unintended consequences of a policy have a detrimental impact on both the economy and society at large.^3^ Based on our findings, other jurisdictions can anticipate this when attempting to introduce tobacco-control policies.

Tobacco companies have been able to disseminate their research to wide audiences in their attempts to increase the public perception that illicit trade is ever increasing, while there remain doubts as to whether this is in fact the case. Even though PMI’s attempts to stop the introduction of standardised packaging in the UK were unsuccessful, news articles in the global media promoting tobacco-industry data on illicit tobacco are still evident. For example, in 2019, media articles containing tobacco-industry data claimed that standardised packaging has led to an increase in illicit trade in Australia and countries seeking to introduce the policy are warned that the same will happen to them.^39 40^

It is incumbent on news publications to question the motives of those presenting them with news stories and transparent media coverage is essential. It is important that the media are aware of the fact that tobacco companies commission research and they must question its validity as this is a tactic replicated globally. Therefore dissemination of these research findings is imperative.

**What this paper adds**

- The threat of an increase in the illicit-tobacco trade is frequently used by tobacco companies to oppose tobacco-control polices, despite allegations of their historical complicity in the trade. Previous research revealed that misleading industry data on illicit tobacco began to appear in UK media immediately after the 2011 announcement of a forthcoming public consultation on standardised packaging.
- Consistent with leaked documents detailing its anti-standardised packaging strategy, Philip Morris International engaged a third party to conduct undercover purchases. The results of the research then led to media coverage which was likely to heighten public consciousness of illicit tobacco and complemented the aim of suggesting that the introduction of standardised packaging would make this worse.
- The articles present findings favourable to the industry, by highlighting counterfeited tobacco and illicit whites where the industry is portrayed as the victim, over tobacco-industry illicit (genuine smuggled product) which points towards potential industry complicity, or, at best, company failure to diligently manage their supply chains.
- One-third of articles containing industry data also mentioned authorities such as Trading Standards, Her Majesty’s Revenue and Customs, police officers and Crimestoppers. Although only a very small proportion of these were true collaborations, the remainder portrayed a sense of partnership between the industry and these authorities, which may have increased the credibility of the tobacco industry’s findings.
- Through engaging a consultant, it appears that Philip Morris International attempted to build relationships with Trading Standards teams in Scotland, offering training and seeking information on where to acquire illicit tobacco. The media frequently reported that data were passed to authorities, yet documents and feedback from some Councils suggest that these data were not necessarily sufficiently detailed for the authorities to take any further action.
- Despite purchases taking place in small specific areas within Scottish Councils, the subsequent media coverage often extrapolated these purchases to the entire town, city or council. Given that test purchasers are suspected to have sought illicit tobacco hotspots, it is highly unlikely that the data acquired from the test purchases were representative.
