# Supplementary material for: Illicit tobacco trade is ‘booming’: UK newspaper coverage of data funded by transnational tobacco companies
Source: Tob Control. 2020 Apr 16;29(e1):e78–86. doi: 10.1136/tobaccocontrol-2018-054902 (PMC7799410; doi:10.1136/tobaccocontrol-2018-054902)
Supplement: Supplementary data [file tobaccocontrol-2018-054902supp001.pdf]

Table S1. Scottish Council trading standards interactions with PMI consultant and press coverage in the Council area following the consultant's UTPs.

Scottish Council	**Response to FOI**	**First known contact between TS/PMI consultant**	**Media coverage**	**Date of media coverage**	**Quote from media article on the scale or nature of illicit trade in the Scottish Council/City/town or other area**
Aberdeen City	Confirmed meeting PMI consultant - Refused access to documents	September 2013	Aberdeen Evening Express	05 March 2014	The North-East is the third worst area in Scotland. [PMI consultant]
Aberdeenshire	Documents released	September 2013	Aberdeen Press & Journal	05 November 2013	…former Scotland Yard detective inspector who has been conducting research into the illegal tobacco trade for the industry, said, since the introduction of standardised packaging for cigarettes in Australia in December 2012, the illicit cigarette trade had risen significantly. (Australia)
			Aberdeen Press & Journal	21 November 2013	Sales of illicit tobacco and cigarettes in the North-East are **rife.**
			Aberdeen Press & Journal	22 January 2014	An investigation by a former Scotland Yard detective has uncovered evidence that the trade in illegal tobacco is **rife** in Inverness.
			Aberdeen Press & Journal	13 February 2015	…nearly a third (30.4%) of all cigarettes consumed in Aberdeen were either contraband, counterfeit or 'illicit whites'…
			Buchan Observer	04 March 2014	Aberdeen, Peterhead and Fraserburgh – is the **third highest** of ten regions across Scotland for locations at which illicit tobacco is sold.
			Ellon Times	04 March 2014	
			Inverurie Herald	04 March 2014	
			Fraserburgh Herald	04 March 2014	
Angus	No record of contact	n/a			
Argyll & Bute	No record of contact	n/a			
Stirling & Clackmannanshire	Documents released	November 2013	Stirling Observer	22 November 2013	…team was able to buy 44 packs of illegal cigarettes and two 50g quantities of roll-your-own tobacco. During the three day operation, which covered Alloa, Falkirk and Stirling…
				21 February 2014	Like other Scottish towns and cities, Stirling has illicit cigarettes for sale and that's a problem which needs to be tackled.

			Daily Record & Sunday Mail	21 November 2013	…team was able to buy 44 packs of illegal cigarettes and two 50g quantities of roll-your-own tobacco. During the three day operation, which covered Alloa, Falkirk and Stirling…
Comhairle nan Eilean Siar	No record of contact	n/a			
Dumfries & Galloway	Documents released	October 2013	Dumfries & Galloway Standard	27 November 2013	Two unnamed Dumfries pubs and a supermarket have been linked to a **booming** trade in illegal tobacco.
				21 February 2014	His research uncovered a **booming** trade in illicit whites – non-duty paid cigarettes smuggled in the UK – with brands such as Fest and Jin Ling easily obtainable throughout the town as well as sizeable amounts of roll-your-own tobacco.
Dundee City	Documents released	July 2013	Daily Record & Sunday Mail	13 November 2014	International criminal gangs targeted Inverness in their bid to **flood** Scotland with fake tobacco which could seriously damage your health – immediately.
East Ayrshire	No record of contact	n/a			
East Dunbartonshire	No record of contact	n/a			
East Lothian	No record of contact	n/a			
East Renfrewshire	No record of contact	n/a			
Edinburgh	Confirmed meeting & reported intelligence received – refused access to documents	September 2013	Evening News	21 November 2013	Edinburgh is being **flooded** with fake fags by organised Eastern European crime gangs, a former top cop has warned.
				23 January 2014	Illegal cigarettes were bought from **one in five venues** across the Capital as part of an undercover sting.
				04 November 2014	The amount of illegal tobacco on sale in the Capital has more than **doubled** in the last year, according to a new study.
			The Scotsman*	09 March 2014	Tobacco smuggling in Scotland has risen by a third in the past two years…Chief Inspector XXXXXXX found that 9.5 per cent of cigarettes on sale in Scotland were either counterfeit, illicit whites or smuggled brands compared to 7.1 per cent in 2011.
				10 March 2014	
Falkirk	Refused access due to ongoing investigation	Unknown	Stirling Observer**	22 November 2013	As above for Stirling & Clackmannanshire
				21 February 2014	As above for Stirling & Clackmannanshire
			Daily Record & Sunday Mail	21 November 2013	As above for Stirling & Clackmannanshire
Fife	Documents released	November 2013	No Press in the dataset		
Glasgow City	Refused to confirm or deny whether it had contact	Unknown	Glasgow Evening Times	27 November 2013	Glasgow is one of the easiest places in the UK to buy illegal tobacco

				05 November 2015	Potentially dangerous illegal cigarettes are easier to buy in Glasgow than they were a year ago, it was claimed today.
Highland	No record of Contact	n/a			
Inverclyde	Confirmed meeting and reported intelligence received but no formal actions taken. No actual documents provided	October 2013	No press in the dataset		
Mid Lothian	No record of contact	n/a			
Moray	No record of contact	n/a			
North Ayrshire	No record of contact	n/a			
North Lanarkshire	Refused to confirm or deny contact	Unknown	Airdrie & Coatbridge Advertiser	12 February 2014	A nationwide study has found evidence that trade in illicit tobacco and cigarettes is **rife** throughout North Lanarkshire.
Orkney	No record of contact	n/a			
Perth & Kinross	No record of contact	n/a			
Renfrewshire	Documents released	October 2013	Paisley Daily Express	21 November 2013	An investigation into the black market tobacco trade has revealed Paisley is being **flooded** with dodgy cigarettes that are generating funds for organised crime.
				27 February 2014	A study into the trade of illegal tobacco has found evidence that the sale of illicit cigarettes is **rife** throughout Renfrewshire.
				13 February 2015	Paisley is today unmasked as a **'hotspot'** for the illegal tobacco trade. A test purchase exercise, led by a former Scotland Yard detective, found that almost 40 per cent of tobacco being offered in the town's shops, pubs and clubs had been smuggled into the country by crooks who were determined to avoid paying tax. That figure is higher than any other town or city in Scotland, including neighbouring Glasgow, where less than a third of the cigarettes sold were found to be illicit.
Scottish Borders	Documents released including spreadsheet of 7 purchases	October 2013	No press in the dataset		
Shetland Island	No record of Contact	n/a			
South Ayrshire	Documents released	October 2013	Scottish Star	02 December 2013	We've been looking at the illicit market for a number of years now. We've gone to some of the wellrette known hotspots, with the Ayr Sunday Market being in that category. We found that the illicit tobacco trade in Ayr is **booming.** [PMI consultant]
South Lanarkshire	No record of Contact	n/a			

West Dunbartonshire	No record of Contact	n/a			
West Lothian	No record of Contact	n/a			

* National paper printed in Edinburgh;** These articles are the same as those that covered Stirling & Clackmannanshire
